# CircSEC24A upregulates TGFBR2 expression to accelerate pancreatic cancer proliferation and migration via sponging to miR-606

**DOI:** 10.1186/s12935-021-02392-y

**Published:** 2021-12-14

**Authors:** Yankun Chen, Simiao Xu, Xinyuan Liu, Xueyi Jiang, Jianxin Jiang

**Affiliations:** 1 Department of Hepatobiliary Surgery, Renmin Hospital of Wuhan University, 99 Ziyang Road, Wuhan, 430060 Hubei People’s Republic of China; 2 Department of Hepatic-Biliary-Pancreatic Surgery, The Affiliated Hospital of Guizhou Medical University, Guiyang, 550000 Guizhou China; 3 Department of Endocrinology, Affiliated Tongji Hospital, Tongji Medical College, Huazhong University of Science and Technology, Wuhan, 430030, Hubei China

**Keywords:** CircSEC24A, Pancreatic cancer, Proliferation, Metastasis, TGFBR2

## Abstract

**Background:**

Circular RNA (circRNA), producing by special selective splicing, was widely expressed in the cytoplasm of eukaryotic cells as a newly non-coding RNAs. It played different roles in a variety of diseases including cancer and performed different functions. Nonetheless, reports on the specific function of circRNA in pancreatic cancer (PC) were still rarely so far. In particular, the role of circSEC24A in PC remains unclear.

**Methods:**

Real-time fluorescent quantitative PCR was used to evaluate the expression level of circSEC24A in pancreatic cancer tissues and cell lines. Furthermore, we used some functional experiments, such as EDU and Transwell assays, to explore the effects of circSEC24A on the proliferation and invasiveness of pancreatic cancer. Finally, the corresponding relationship among circSEC24A, miR-606 and TGFBR2 was explored by dual luciferase reporter and other mechanism studies.

**Results:**

The expression of circSEC24A in both pancreatic cancer tissues and cell lines was evidently up-regulated. Furthermore, knockdown of circSEC24A significantly inhibited the proliferative, migration and invasive capacity of pancreatic cancer cells, whereas miR-606 inhibitor obviously counteracted these effects. Further study confirmed that circSEC24A alleviated suppression on target TGFBR2 expression by directly sponging miR-606 and then influenced the tumorigenesis of pancreatic cancer.

**Conclusions:**

These findings indicated that the progression of pancreatic cancer can be driven by circSEC24A influencing miR-606/TGFBR2 axis. Therefore, circSEC24A might be used as a critical biomarker influencing the early diagnosis and prognosis of pancreatic cancer.

**Supplementary Information:**

The online version contains supplementary material available at 10.1186/s12935-021-02392-y.

## Background

Although pancreatic cancer is a relatively rare form of cancer in digestive system, the increase in the morbidity of pancreatic cancer is continually developed [1]. Pancreatic cancer is a dismal disease with a poor prognosis and a 5-year survival rated even far lower than 7%, regardless of appropriate treatment [2]. The main therapy of pancreatic cancer is a radical surgical resection, which only applies to a small number of patients, because of lacking obvious clinical symptoms in early stage, the relatively rapid progression and the early development of systemic metastasis [3]. In addition, pancreatic cancer displays high resistance to Gemcitabine plus cisplatin chemotherapy, resulting in high recurrence rate of pancreatic cancer [4]. Hence, it is necessary to explore novel biomarkers for early diagnosis of pancreatic cancer and seek potential therapeutic targets for pancreatic cancer.

Circular RNAs (circRNAs) can be covalently linked at the 5′ and 3′ ends to form a circular structure. As a new type of non-coding RNA, it has not been found to have protein-coding ability [5]. CircRNAs are highly conserved and abundant with closed-loop molecular structures, which are not affected by exonuclease and are not easy to be degraded [6]. There is increasing evidence that circRNAs participate in multiple biological functions in tumors, including cell proliferation, apoptosis, differentiation, metastasis and regulation of gene expression [7]. CircRNAs have miRNA response elements (MREs), hence, circRNAs can be utilized as competing endogenous RNA (ceRNA) to indirectly modulate gene expression in posttranscriptional level via specially binding to miRNAs [8]. Zhou et al. [9] reported that circFAT1 promoted malignant progression of cervical cancer by binding miR-409-3p to upregulate CDK8 and activating ERK1/2 signaling. Gu et al. [10] found that circHIPK3 resulted in activation of AKT/mTOR signaling to facilitate lung cancer cell growth, metastasis and glycolysis via serving as sponge for miR-381-3p. Previous study suggested that circCDR1as was overexpressed in pancreatic cancer and promoted the proliferation, migration and invasion of pancreatic cancer cells by regulating E2F3 expression via functioning as sponge for miR-432-5p [11]. In addition, inhibitory expression of circ_0013912 significantly suppressed pancreatic cancer cell proliferation and metastasis via sponging miR-7-5p [12]. Zhang et al. declared that circ_001587 elevated SLC4A4 expression to suppress malignant phenotype of pancreatic cancer, including angiogenesis and metastasis. The underlying mechanism was that circ_001587 competitively sponged to miR-223, thereby increased the expression of SLC4A4, which was a tumor suppressor [13]. Therefore, deep exploration of the circRNA-miRNA-mRNA network for pancreatic cancer is benefited for excavating novel biomarkers for diagnosis and treatment of pancreatic cancer.

In this study, we identified a novel circRNA hsa_circ_0003180 (circSEC24A) with 252 nucleotides in length, which was derived from back-splicing the SEC24A mRNA and located on chromosome 5: 134002512–134007576. Interestingly, circSEC24A was overexpression in pancreatic cancer tissues and high expression levels was positively correlation to malignant phenotype, including cell proliferation and metastasis. Mechanistically, circSEC24A acted as a ceRNA of TGFBR2 to bind miR-606, blocking the inhibitory function of miR-606 on TGFBR2. Therefore, circSEC24A/miR-606/TGFBR2 signaling axis might be one of the indispensable factors affecting the therapeutic effect of pancreatic cancer.

## Materials and methods

### Clinical tissue specimens

All clinical specimens including pancreatic cancer patients’ tissues and matched adjacent normal tissues were obtained from 20 patients with pancreatic cancer surgically resected in the Renmin Hospital of Wuhan University. The patients without radiotherapy, chemotherapy or other neoadjuvant treatments were selected for our study. The group selected as normal tissues showed no cancer cells in pathological examination. After surgical resection, the specimens from those patients were collected immediately. This research gained permission from the Ethics Committee of Renmin Hospital of Wuhan University.

### Reverse transcription-quantitative PCR (RTqPCR)

The TRIzol® reagent (No. R701-01, Vazyme Biotech Co., Ltd) in our study was used to isolate the total RNA from cultured cells or frozen tissues. The purity (OD260/280) and concentration of RNA was detected using NanoDrop 2000 instrument (Invitrogen, USA). For circRNA and mRNA, the complementary DNA (cDNA) was generated using the PrimeScript RT Master Mix (No. R323-01, Vazyme Biotech Co., Ltd) with random primers. For miRNA, the cDNA was generated using the PrimeScript RT Reagent Kit (No. MR101-01, Vazyme Biotech Co., Ltd) with specific stem-loop primers. Subsequently, the resulting cDNA was used for qPCR using the Realtime PCR Master Mix (No. Q711-02, SYBR Green, Vazyme Biotech Co., Ltd) in CFX Connect Fluorescent Quantitative PCR Instrument (Bio-Rad, USA). Various primers (RiboBio, Guangzhou, China) used for qPCR were listed below. circSEC24A F (forward): 5′-TTCAGCTGTCAACCAAGAAGGT-3′ and R (reverse): 5′-AAAGTTGTAGGCAGAGGTGGA-3′); miR-606 F: 5′-CGCGCGAAACTACTGAAAATC-3′ and R: 5′-AGTGCAGGGTCCGAGGTATT-3′; GAPDH F: 5′-GGAGCGAGATCCCTCCAAAAT-3′ and R: 5′-GGCTGTTGTCATACTTCTCATGG-3′; U6 F: 5′-CTGGCTTCGGCAGCACA-3′ and R: 5′-AACGCTTCACGAATTTGCGT-3′; TGFBR2 F: 5′-GTAGCTCTGATGAGTGCAATGAC-3′ and R: 5′-CAGATATGGCAACTCCCAGTG-3′. The reference standard for circSEC24A and TGFBR2 was GAPDH. Specially, the reference standard for miR-606 was U6.

### RNase R treatment

RNase R was an exonuclease that primarily acted on and removed linear RNA. To digest the linear RNA from PANC-1 and MIA PaCa-2, total isolated RNA was mixed with RNase R (30 units, Shanghai Hengfei Biotechnology Co., Ltd.) for 30 min at 37 °C. Then, real-time PCR was performed to quantitative the expression of circSEC24A and SEC24A, respectively.

### Fluorescence in situ hybridization

FISH, an important non-radioactive in situ hybridization technique, was used in our study to explore the location of circSEC24A in pancreatic cancer cells. The special circSEC24A probes, which working concentration was 0.5 µM, were purchased from Ruibo Biotechnology (Guangzhou, China). Then, the hybridization was executed all night with the probes. Finally, we utilized a fluorescence microscope (Olympus, Japan) to capture the images and selected some representative images for analysis.

### Subcellular fractionation

About 2 × 10^6^ PANC-1 cells were collected and rinsed with cold PBS. Nuclear and cytoplasmic RNA were collected respectively using the PARIS™ kit (Invitrogen, USA) according to the manufacturer’s manual. Subsequently, circSEC24A expression was analyzed by qRT-PCR, meanwhile, 18S rRNA and U6 detected as control of cell cytoplasm and cell nuclei, respectively.

### Cell culture and transfection

Normal pancreatic duct epithelial cell line HPDE, and 4 types of pancreatic cancer cell lines (PANC-1, MIA PaCa-2, SW1990, BxPC-3) obtained from American Type Culture Collection (ATCC) were indispensable members of our various experiments. HPDE and BxPC-3 were maintained in RPMI 1640 (No. A1049101, Gibco, USA); while other cell lines including PANC-1, MIA PaCa-2 and SW1990 were maintained in high glucose DMEM (No. 11965092, Gibco, USA). To maintain cells viability, not only 10% fetal bovine serum (FBS, No. 10099141, Gibco, USA) but 1% penicillin-streptomycin solution (No. 15070063, Gibco, USA) should be added to the medium. The medium was exposed to UV radiation for 30 min before we used. In conclusion, all cells we used were maintained and stored following the instructions provided by ATCC. Moreover, the appropriate temperature (37 °C) and the suitable concentration of carbon dioxide(5% CO_2_)can effectively promoted cell growth rapidly, so all cells need to be grown in special incubators that meet these conditions.

Firstly, we seeded PANC-1 and MIA PaCa-2 cells into 6-well plates. Transfection can be carried out when cells attached to the bottom of the hole and 60–70% of the total area is reached. Different reagents were subsequently transfected into different cell lines, respectively. These reagents were synthesized by Ruibo Biotechnology (Guangzhou, China) and included si-circSEC24A, miR-606 mimics, miR-606 inhibitors, and negative controls. Appropriate amount of Lipofectamine™3000 reagent (No. L3000015, Invitrogen, USA) was added during the transfection process to improve the transfection efficiency. Additionally, we purchased sh-circSEC24A and sh-NC lentivirus vector from Ruibo Biotechnology (Guangzhou, China), then transfected into PANC-1 cells for lentiviral packaging according to the manufacturer’s protocol.

### Cell counting Kit8 assays

About 2000 transfected cells were plated and cultured until adherent. Then, original CCK-8 solution (Boster, China) was mixed with culture medium according to the rate of 1:9 and added directly into each well at the indicated time. After 2 h of incubation, we utilized the optional density (OD) value measured by microplate reader (BioTek, USA) to evaluated cell proliferation ability.

### Colony formation assays

About 500 transfected cells were seeded in 6-well plates. Two weeks later, the clones visible to the naked eye appeared. After washing with PBS (Gibco, USA) for three times, we used 1 mL 4% paraformaldehyde (Vazyme Biotech Co., Ltd) to fix cells for 30 min. In order to stain the cells, it was subsequently maintained in 0.1% crystal violet solution (Vazyme Biotech Co., Ltd) for another 30 min. Excess staining was removed by washing with PBS solution and cell counts were performed with a clear field of vision.

### EDU assays

The EDU cell Proliferation Kit (No. C0075S, Beyotime Biotechnology, China) was used to assess the proliferation ability of cells. Cover glass slides were added into the six-well plate and pancreatic cancer cells with different treated were planted. Before starting the experiment, it is a priority to configure the relevant working solution including EDU working solution. The cells in each well were then immersed in a new EDU working solution to allow the EDU to penetrate the DNA while it was being replicated. Then, the cells were fixed and stained according to the manufacturer’s protocol. Subsequently, we utilized a fluorescence microscope (Olympus, Japan) to acquire the images. The data obtained above are processed by Photoshop software (version 21.0).

### Flow cytometry analysis

Transduced cells (at least 1 × 10^6^) were trypsinized and then washed in pre-cooled PBS. Cell fixation was conducted using 70% ethanol. Then, the cells were stained using PI and RNase reagent (Si Nan Biotechnology Services Co., LTD, China) and incubated at 37 °C. Cell cycle distribution analysis was performed using a flow cytometry device (BD Biosciences, USA).

### Wound healing assays

Pancreatic cancer cells were cultured in 6-well plates until to 100% confluence. Next, a straight line of scars was generated via a tool such as sterile pipette device used to scratch the cell monolayer. The cell scratch wound was washed with PBS and treated with serum depleted media. The random injury filed of cell layer was photographed by a fluorescence microscope at 0 and 24 h. Representative images we screened were analyzed via GraphPad and then calculated the relative migration rate.

### Transwell migration and invasion assays

The matrigel mix (BD Biosciences, USA) need first paved at the surface of upper chamber for invasion assays. In contrast, the migration experiment did not require the application of matrigel mix. After the relevant items have been prepared, 200 µL serum-free medium containing 5 × 10^4^ pancreatic cancer cells was added slowly into upper chamber (Corning, USA). Then, 700 µL of the culture medium with 10% FBS was added into the bottom chamber. The chambers were fixed and then stained after migration for 28 h or invasion for 36 h. Residual staining solution and unpenetrated cells were removed with PBS and cotton swabs, respectively. After nature air dries, the migratory or invasive cells were photographed and counted via fluorescence microscope.

### Western blot

The protein for subsequent detection was extracted from the previously transfected cells under the action of Radio Immunoprecipitation Assays lysis buffer (RIPA, Boster, China). The amounts of protein were assessed by bicinchoninic acid (BCA, Boster, China). Owing to the different molecular weights of the proteins being measured, they can be isolated and formed into bands in the 10% SDS-PAGE. The isolated proteins were then transferred to polyvinylidene fluoride (PVDF) membranes (Boster, China). The PVDF membranes was incubated with 5% skim milk. The cut membranes were then placed in the primary antibodies, respectively. The primary antibodies purchased from Abcam (MA, USA) were as following: anti-CDK6, anti-cyclin D1, anti-CDK4, anti-GAPDH, anti-ZEB2, anti-Snail, anti-Twist, anti-TGFBR2, anti-AKT, anti-p-AKT, anti-ERK, anti-p-ERK, anti-smad2, anti-p-smad2. All antibodies were diluted in skim milk at the concentration of 1:1000. The membranes were immersed completely in secondary antibody (abcam, USA) at room temperature. The blots acquired in our study were dripped with ECL chemiluminescent reagent (Servicebio, Wuhan, China) to produce a light-emitting reaction that can be captured and visualized by the ChemiDoc XRS+ (Bio-Rad), allowing us to analyze each blot with Image Lab Software.

### Bioinformatics analysis

GEO database (GSE69362) was performed to screen for significantly differentially expressed circRNAs in pancreatic cancer. This dataset containing circRNA profiling in six pairs of human PDAC and adjacent normal tissue was conducted by microarray. The CSCD database, TargetScan database and other databases were used to explore the possible role network of circSEC24A in pancreatic cancer.

### Luciferase reporter assay

About 3 × 10^5^ cells were seeded into each well of 24-well plates. To construct respectively the special plasmids, the WT and Mut 3′UTRs of circSEC24A or TGFBR2 were synthesized by Ruibo Biotechnology (Guangzhou, China), termed as circSEC24A-WT, circSEC24A-Mut, TGFBR2-WT and TGFBR2-Mut. Then, the plasmids obtained above were co-transfected into cells in pairs using Lipofectamine^TM^3000 reagent (Invitrogen). 48 h later, the luciferase activities were examined in line with the manufacturer’s manual.

### Xenograft tumorigenesis

The PANC-1 cells used in our experiment were treated with sh-circSEC24A or sh-NC lentivirus. Next, 5 × 10^6^ treated PANC-1 cells suspension was prepared and then subcutaneously injected into 6-week-old female BALB/c nude mice (Beijing Laboratory Animal Center, Beijing, China). Next, we randomly divided those mice into circSEC24A silenced group and control group. Tumor volume in each mouse was monitored weekly. Finally, mice were sacrificed and subcutaneous tumor were detected for tumor weight and IHC staining.

### Immunohistochemical (IHC) staining

Tissue samples were fixed in 4% paraformaldehyde and embedded in paraffin. Paraffin sections were dewaxed and re-hydrated in ethanol in descending concentrations (100%, 90%, 80%, 70%). The tissue sections were incubated with anti-TGFBR2, anti-PCNA and anti-Ki-67 (1:200, Abcam, USA) primary antibodies at 4 °C overnight and then incubated with secondary antibody.

### Statistical analysis

GraphPad Prism (version 8.0.1) was used for statistical analysis in our study. All Data we acquired in these experiments were presented as the mean ± SD. Only *P *< 0.05 indicates that the difference is statistically significant.

## Results

### CircSEC24A is highly expressed in pancreatic cancer

**Fig. 1:**
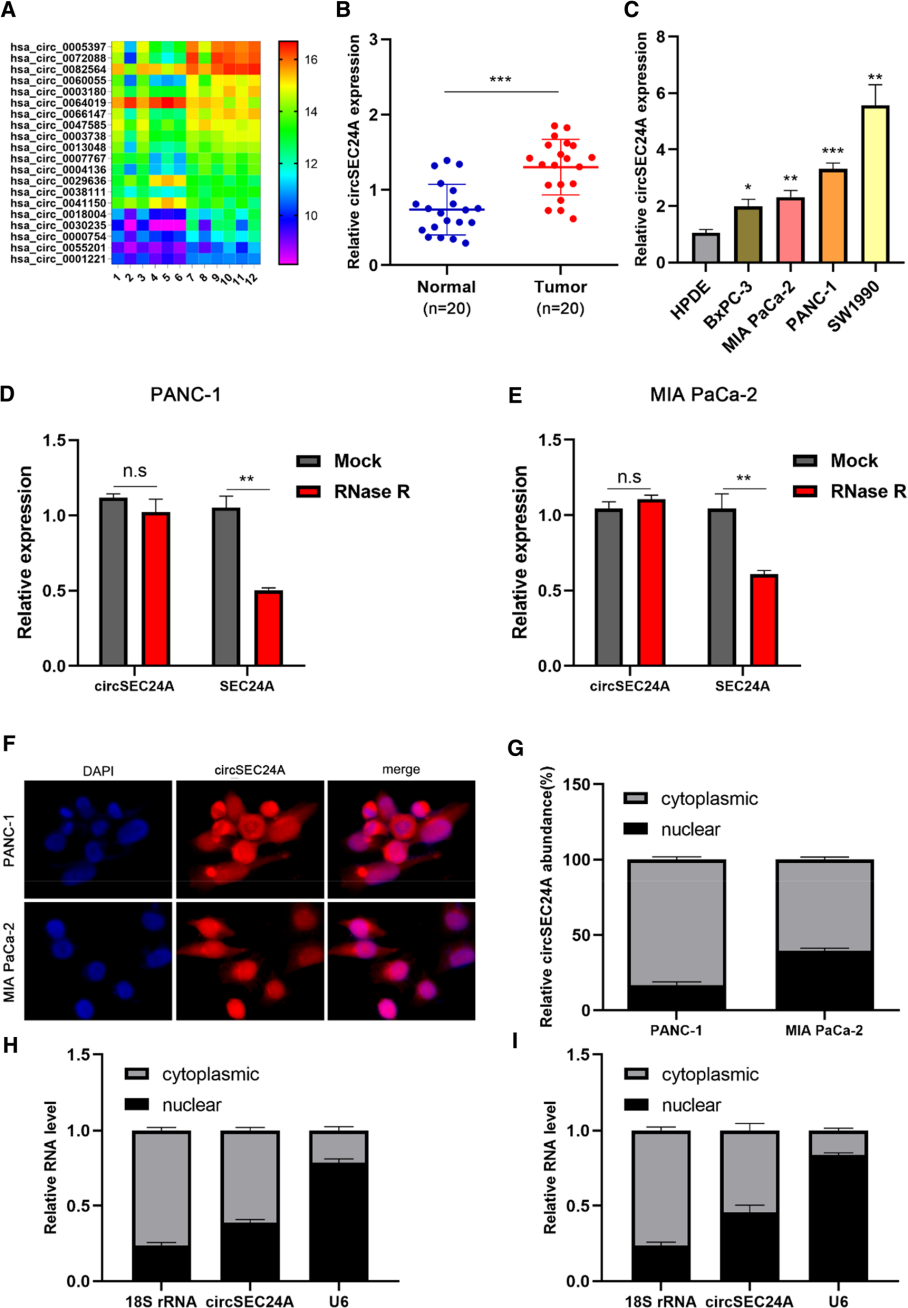
CircSEC24A was highly expressed in pancreatic cancer tissues and cells. **A** The circRNAs with significantly differentially expressed were listed by heatmap. **B**, **C** The expression of circSEC24A in pancreatic cancer tissues (n = 20) and cells. **D**, **E** The mRNA expression of circSEC24A and SEC24A mRNA in PANC-1 and MIA CaPa-2 cell lines was detected by qPCR. **F**–**I** FISH and subcellular fraction assays showed subcellular location of circSEC24A. Representative images were obtained at ×400 magnification

To explore the expression profiles of circRNA in pancreatic cancer, we performed bioinformatics methods to screen differentially expressed circRNA based on GEO database (https://www.ncbi.nlm.nih.gov/geo/, GSE69362) via R software using “limma” and “heatmap” packages. Top 20 differentially expressed circRNA was list in Fig. 1A (Additional file 1). Through reviewing literature and measuring the expression in pancreatic cancer tissues, we filtered out hsa_circ_0003180 (circSEC24A) as our candidate circRNA for subsequent research. Firstly, PCR analysis suggested that circSEC24A was significantly highly expressed in pancreatic cancer tissues compared with their paired adjacent normal tissues (Fig. 1B). Next, we measured the expression of circSEC24A in different pancreatic cancer cell lines compared with human pancreatic duct epithelial cells (HPDE). Surprisingly, four different pancreatic cancer cell lines were all higher expression than HPDE (Fig. 1C). To detect the stability of circSEC24A, we utilized RNase R treatment to digest circSEC24A and the mRNA of SEC24A respectively. The results revealed that circSEC24A was not able to be digested by RNase R, while the SEC24A mRNA expression dramatically decreased after treating with RNase R (Fig. 1D, E). FISH and subcellular fraction assays were performed to measure the subcellular localization of circSEC24A, the results confirmed that circSEC24A was predominantly located in the cytoplasm, which supported the hypothesis that circSEC24A might serve as molecular sponge for miRNA to indirectly regulate gene expression in post-transcriptional levels (Fig. 1F–I).

### CircSEC24A facilitates pancreatic cancer cell proliferation

**Fig. 2:**
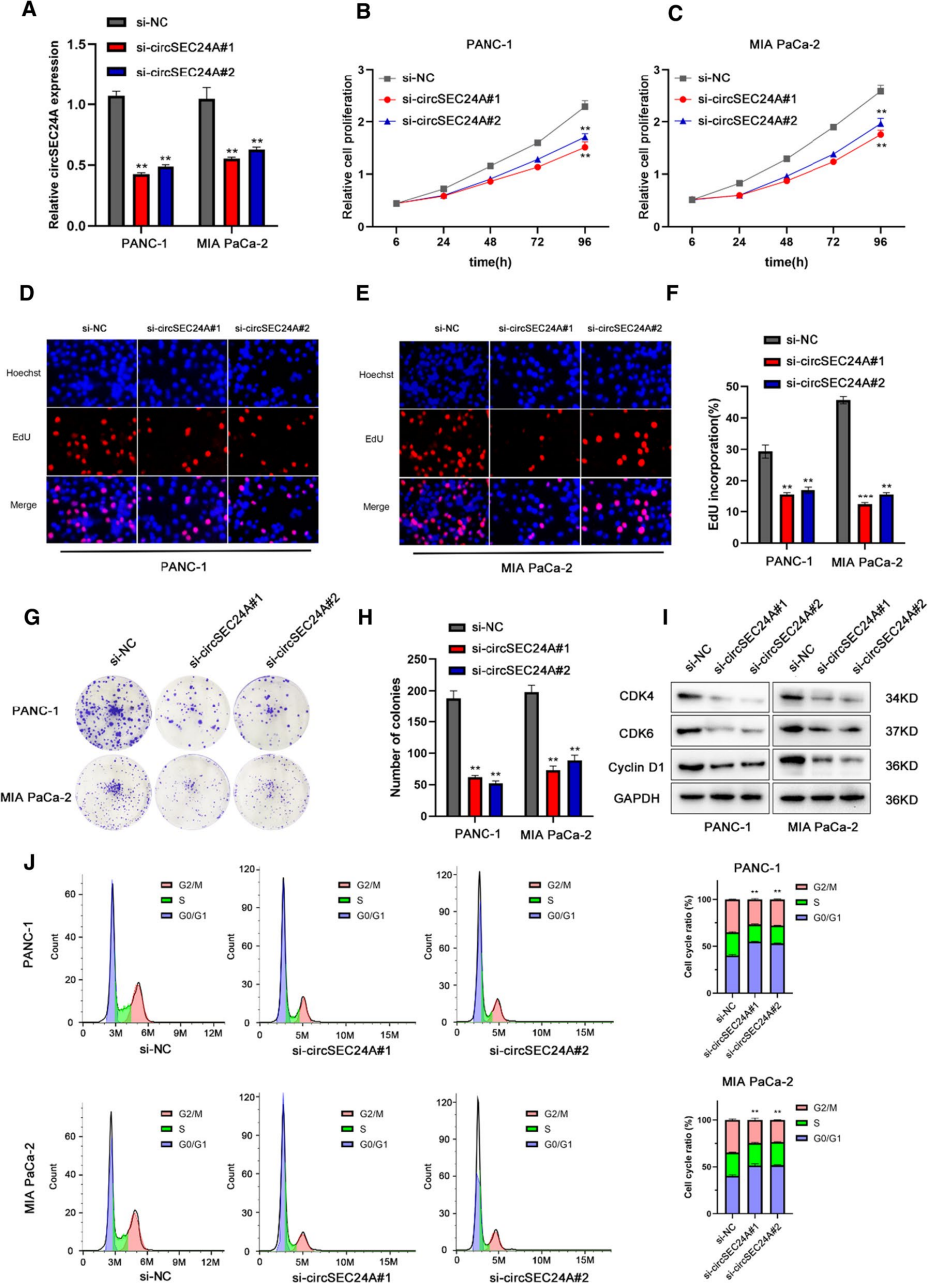
CircSEC24A significantly promoted the proliferation of pancreatic cancer cells. **A** The circSEC24A expression in PANC-1 and MIA CaPa-2 cells transfected with si-circSEC24A by qRT-PCR. **B**, **C** CCK-8 assays was used to detected the proliferation ability of pancreatic cancer cells after different treatments. **D**–**F** EDU assays evaluated the proliferation of cells after silencing circSEC24A expression. Representative images of were obtained at ×400 magnification. **G**, **H** Colony formation assays was performed to count and analysis the number of cells colony formations. **I** The expression of CDK4, CDK6 and Cyclin D1 in pancreatic cancer cells were detected by western blot. **J** Flow cytometry was used to analyse the effects of circSEC24A on PANC-1 and MIA CaPa-2 cell cycle progression

To detect the proliferated ability of circSEC24A in pancreatic cancer cells, PANC-1 and MIA PaCa-2 were transfected with silencing sequences and negative control sequence, respectively. PCR analysis was performed to evaluate the effectiveness of transfection. The results indicated that silencing sequences was successfully transfected into pancreatic cancer cells and downregulated the circSEC24A expression effectively (Fig. 2A). CCK-8, EDU and colony formation assays were respectively evaluated the proliferation ability from different angles. CCK-8 assays suggested that silencing circSEC24A expression dramatically suppressed cell ability compared with control groups (Fig. 2B, C). In addition, EDU assays illustrated that silencing circSEC24A expression significantly inhibited cell growth (Fig. 2D–F). Moreover, colony formation assays indicated that negative control groups displayed more numbers of colony formations than silence groups (Fig. 2G, H). Eventually, we utilized western blot to detect expression of CDK4, CDK6 and Cyclin D1, which were key proteins in transition of cell cycle from G1 to S phase. The results demonstrated that silencing circSEC24A expression resulted in protein downregulation of CDK4, CDK6 and Cyclin D1, indicating that inhibited circSEC24A could negatively modulate pancreatic cancer cell proliferation (Fig. 2I). In PANC-1 and MIA CaPa-2 cells with low circSEC24A expression level, the proportion of G0/G1 phase cells was increased, and the proportion of S and G2/M phase cells was decreased (Fig. 2J).

### CircSEC24A facilitates pancreatic cancer migration and invasion

**Fig. 3:**
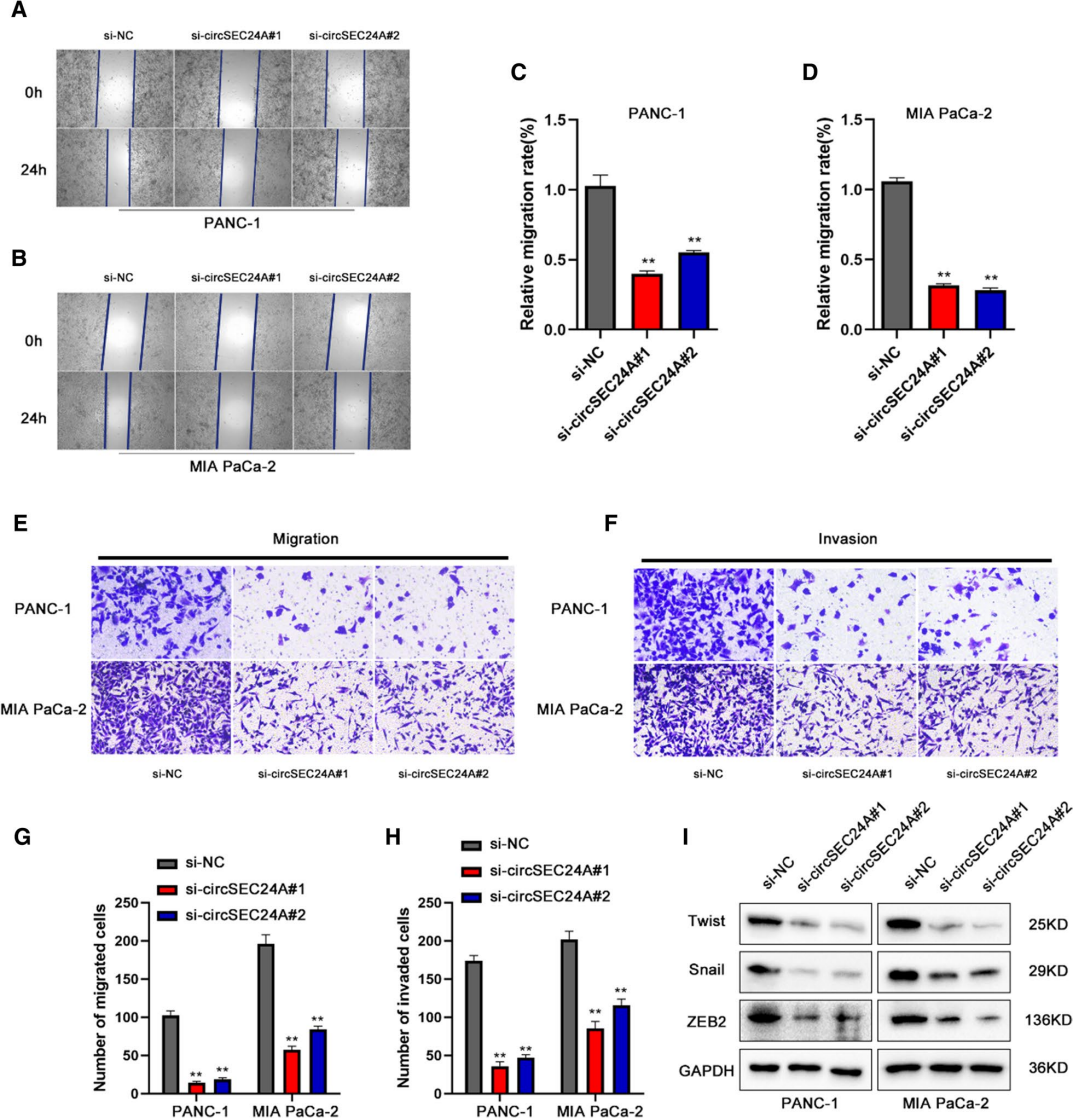
Downregulation of circSEC24A results in suppression of pancreatic cells migration and invasion. **A**, **C** Migration in PANC-1 cells detected using wound healing assays. **B**, **D** Migration in MIA CaPa-2 cells detected using wound healing assays. **E**, **G** Cell migratory capabilities were assessed by transwell assays. **F**, **H** Cell invasive capabilities were assessed by transwell assays. Representative images were obtained at ×200 magnification. **I** Western blot was performed to measure the protein markers of EMT

To further confirm the effect of circSEC24A on migration and invasion in pancreatic cancer cells, transwell assays and wound healing assays were performed to measure the migrated and invaded ability. Wound healing assays illustrated that the migrated ability of silencing circSEC24A expression groups was constraint compared with control group (Fig. 3A–D). Transwell assays further verified that the cell migration and invasion was also constraint with or without Matrigel in silencing circSEC24A expression groups (Fig. 3E–H). Subsequently, we performed western blot to measure the protein markers of epithelial to mesenchymal transition (EMT), including Twist1, Snail1 and ZEB2. Interestingly, knockdown expression of circSEC24A obviously inhibited the protein levels of Twist1, Snail1 and ZEB2, suggesting circSEC24A had the ability to promote EMT transition (Fig. 3I).

### CircSEC24A directly binds to miR-606 to mediate proliferation and migration of pancreatic cancer cells

**Fig. 4:**
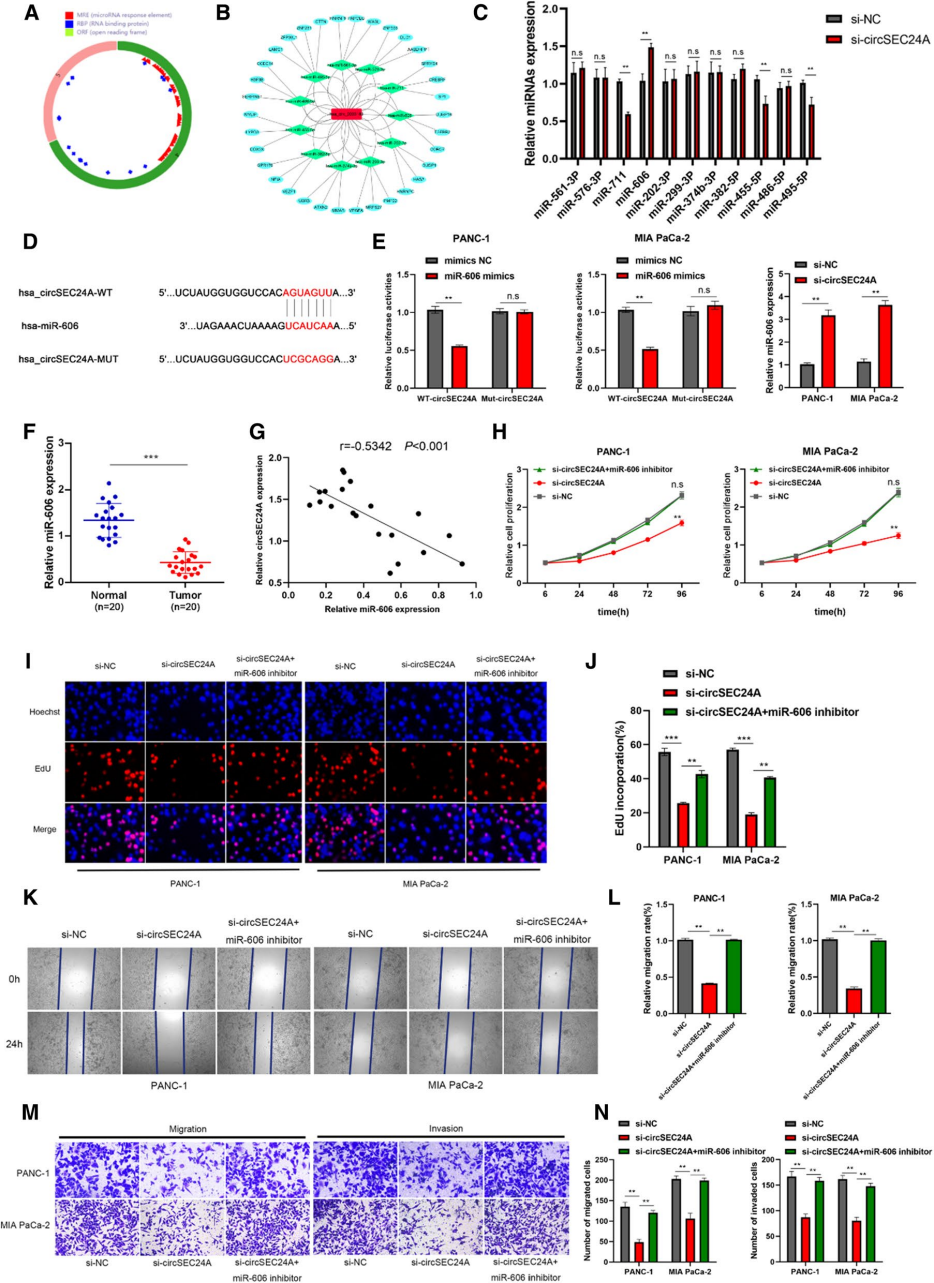
CircSEC24A directly binds to miR-606 to mediate proliferation and migration of pancreatic cancer cells. **A** The schematic diagram of circSEC24A which contains MER. **B** CircRNA-miRNA-mRNA network was performed to predict the potential miRNA and mRNA based on CSCD database. **C** PCR analysis was performed to confirm the potential binding miRNA. **D** The schematic diagram showed the wild type binding sequence and the mutant type binding sequence of circSEC24A with miR-606. **E** Dual luciferase reporter assay was performed to confirm the binding site. **F** The expression of miR-606 was detected by PCR analysis. **G** The correlation analysis between circSEC24A and miR-606. **H**–**J** Rescue experiments was performed to measure the effect of miR-606 inhibitor combining with si-circSEC24A on proliferation through CCK-8 and EDU assays. Representative images were obtained at ×400 magnification. **K**–**N** Rescue experiments was performed to measure the effect of miR-606 inhibitor combining with si-circSEC24A on migration and invasion through transwell and wound healing assays. Representative images were obtained at ×200 magnification

Previous experiment results showed circSEC24A mainly concentrated on the cytoplasm, indicating circSEC24A might have potential to bind to miRNA. Subsequently, we confirmed that MRE widely existed in circSEC24A based on CSCD database (http://gb.whu.edu.cn/CSCD/) (Fig. 4A). CircRNA-miRNA-mRNA network was constructed with predicted target of miRNA and mRNA based on CSCD database, Circular RNA Interactome database (https://circinteractome.irp.nia.nih.gov/index.html), miRDB (http://mirdb.org/), miRTarbase (http://mirtarbase.cuhk.edu.cn/php/index.php) and Targetscan (http://www.targetscan.org/vert_71/) by using cytoscape software (Fig. 4B). There were 11 potential binding miRNAs and we performed PCR analysis to measure their expression in control groups and silencing groups, respectively. The results suggested that 4 of them displayed differential expression and only miR-606 showed a negative correlation with circSEC24A expression, hence, we selected miR-606 as the downstream target (Fig. 4C). To further confirm whether circSEC24A could exert as a sponge for miR-606, dual luciferase reporter assay was performed to measure the binding between circSEC24A and miR-606. The prediction binding site was displayed in Fig. 4D. Dual luciferase reporter assay suggested that miR-606 overexpressed significantly inhibited wild type circSEC24A luciferase reporter activity. However, mutant type circSEC24A luciferase reporter activity seemed to change little (Fig. 4E). PCR analysis indicated silencing circSEC24A expression dramatically increased expression of miR-606 (Fig. 4F). Additionally, we detected miR-606 expression in pancreatic cancer tissues and adjacent normal tissues by using PCR analysis, revealing that miR-606 was low expression in tumor tissues (Fig. 4G). Pearson correlation analysis showed that circSEC24A was negatively correlated with miR-606 (Fig. 4H). Subsequently, rescuing experiments were performed to further evaluate the effect of miR-606 on proliferation, migration and invasion in pancreatic cancer cells. The results illustrated that inhibited expression of miR-606 in silencing circSEC24A groups resulted in accelerating effect on proliferation and migration, partly reversing the silencing circSEC24A expression mediated inhibitory function (Fig. 4I–O).

### TGFBR2 is target of miR-606 and promotes pancreatic cancer cell proliferation and migration

**Fig. 5:**
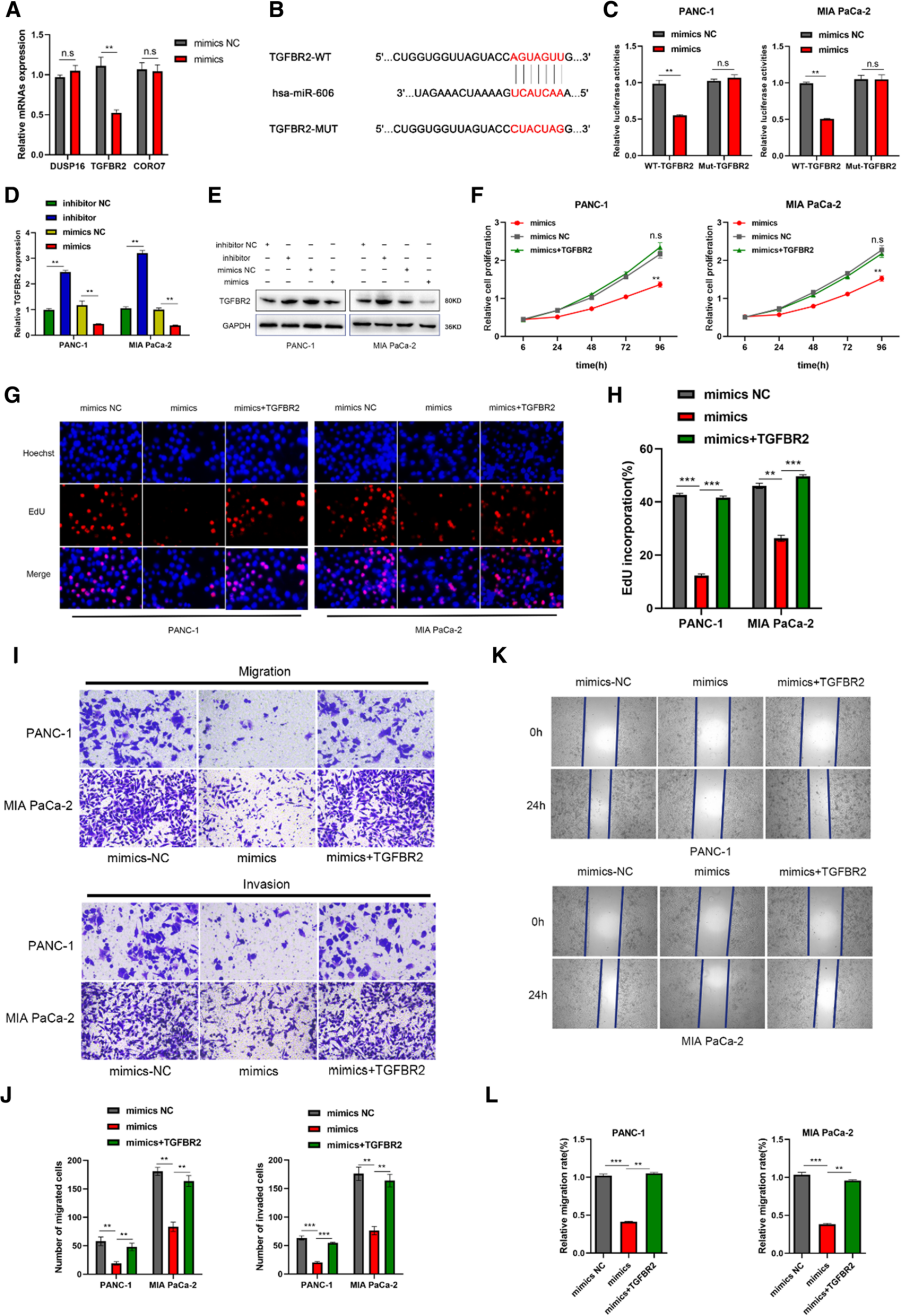
TGFBR2 is target of miR-606 and promotes pancreatic cancer cell proliferation and migration. **A** The candidate target of miR-606 was identified by PCR analysis. **B** The schematic diagram showed the wild type binding sequence and the mutant type binding sequence of TGFBR2 with miR-606. **C** Dual luciferase reporter assay was performed to confirm the binding site. **D** PCR analysis was performed to measure the mRNA expression level of TGFBR2 in pancreatic cancer cells transfected with miR-606 mimics or inhibitors. **E** Western blot was performed to measure the protein expression level of TGFBR2 in pancreatic cancer cells transfected with miR-606 mimics or inhibitors. **F**–**H** Rescue experiments was performed to measure the effect of miR-606 mimics combining with TGFBR2 on proliferation through CCK-8 and EDU assays. Representative images were obtained at ×400 magnification. **I**–**L** Rescue experiments was performed to measure the effect of miR-606 mimics combining with TGFBR2 on migration and invasion through transwell and wound healing assays. Representative images were obtained at ×200 magnification

Previous prediction results indicated miR-606 had three potential targets (DUSP16, TGFBR2 and CORO7) acquired by Venn analysis from miRDB, miRTarbase and Targetscan database. Firstly, PCR analysis was performed to measure the expression changes of these three potential targets in miR-606 overexpression groups and control groups, respectively. The results indicated that only TGFBR2 was downregulated in miR-606 overexpression group, suggesting that TGFBR2 might be a downstream target of miR-606 (Fig. 5A). Subsequently, we utilized dual luciferase reporter assay to further verify the interaction between miR-606 and TGFBR2. The wild type and mutant type sequences of TGFBR2 were shown in Fig. 5B. Dual luciferase reporter assay illustrated that miR-606 overexpression dramatically decreased the luciferase activity in wild type TGFBR2 but not changes in mutant type TGFBR2, suggesting that miR-606 could directly bind to the 3’UTR of TGFBR2 mRNA (Fig. 5C). In addition, PCR analysis and western blot both confirmed that upregulated or downregulated miR-606 expression resulted in inhibitory or facilitating TGFBR2 expression in mRNA levels or protein levels, respectively (Fig. 5D, E). Gain-function experiments were performed to evaluate the effect of TGFBR2 on miR-606 mediated inhibitory function on proliferation, migration and invasion. As expected, the increasing TGFBR2 expression in miR-606 overexpression groups could partly rescue the miR-606 overexpression mediated inhibitory function on proliferation, migration and invasion (Fig. 5F–L). These results further confirmed that TGFBR2 was the target of miR-606.

### CircSEC24A promotes TGFBR2 expression via sponging to miR-606 and activates AKT signaling

**Fig. 6:**
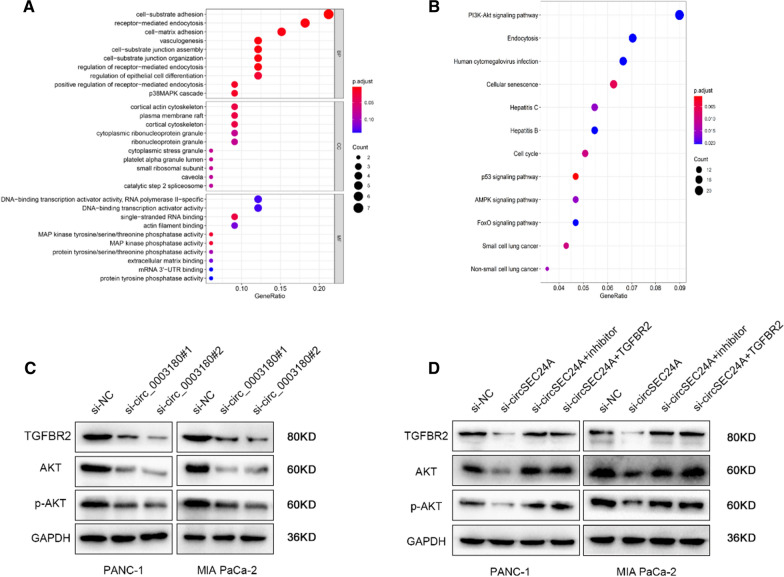
CircSEC24A promotes TGFBR2 expression via sponging to miR-606 and activates AKT signaling. **A** Go enrichment analysis was performed to explore the potential function on cellular component (CC), molecular function (MF) and biological process (BP), respectively. **B** KEGG enrichment analysis was performed to explore the potential signaling pathway correlated with CircSEC24A. **C** Western blot was performed to confirm the result of KEGG enrichment (PI3K/AKT signaling pathway). **D** Western blot was performed to further evaluate the key protein expression levels of AKT signaling pathway. Then miR-606 inhibitor or TGFBR2 vector was respectively cultured with si-circSEC24A pancreatic cancer cells. The signaling pathway markers was measured by western blot

To further explore the underlying molecular mechanism how circSEC24A promotes malignant phenotype, we utilized the top 200 circSEC24A-related genes for GO and KEGG enrichment analysis via R software using “clusterProfilerGO” and “clusterProfilerKEGG” packages. Interestingly, GO enrichment analysis illustrated that circSEC24A was significantly associated with cell adhesion, receptor-mediated endocytosis, actin filament binding and MAPK phosphatase activity, which all supported for cell proliferation, metastasis and signaling transduction (Fig. 6A). KEGG enrichment analysis also illustrated that circSEC24A was obviously correlated with PI3K-AKT signaling pathway, endocytosis and cell cycle et al. (Fig. 6B). Subsequently, we performed western blot assay to confirm the enrichment results. First, we detected expression of TGFBR2 in silencing circSEC24A expression groups, indicating TGFBR2 was significantly downregulated in silencing groups (Fig. 6C). Meanwhile, total AKT and phosphorylated AKT were also downregulated in silencing circSEC4A expression groups (Fig. 6C). Additionally, we added TGFBR2 overexpressed vectors or miR-606 inhibitors to circSEC4A silencing group and measured these protein expression levels. The results indicated that silencing circSEC4A expression led to downregulation of these protein, however, TGFBR2 overexpression or miR-606 downregulation could partly increase the protein expression of circSEC24A mediated inhibitory effect (Fig. 6D). These results suggested circSEC24A could activate AKT signaling and at least these effects partly depended on circSEC24A sponging to miR-606 to modulate TGFBR2 expression.

### CircSEC24A promotes the growth of pancreatic cancer cell in vivo

**Fig. 7:**
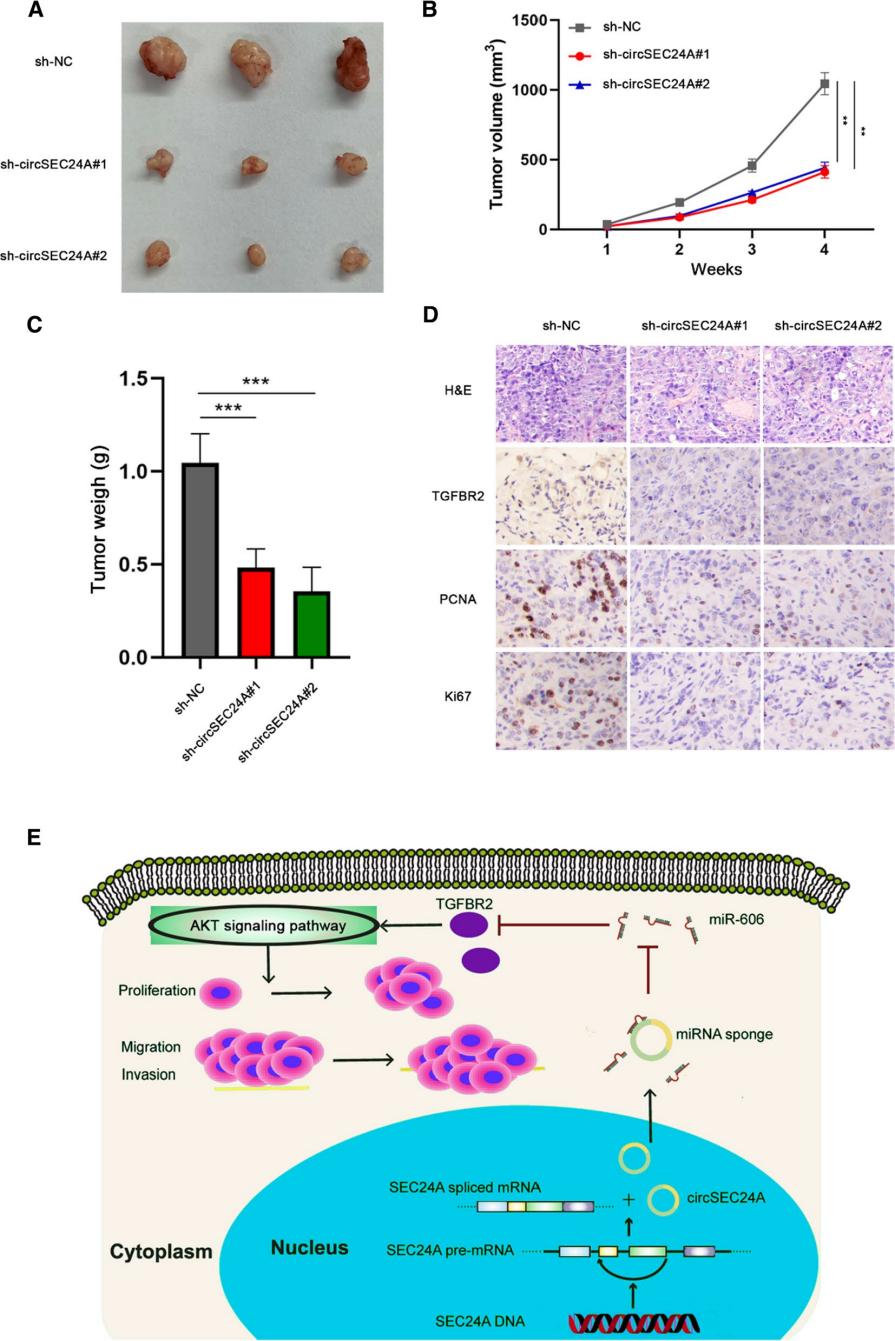
CircSEC24A promotes the progression of pancreatic cancer in vivo. **A**–**C** Tumor size and weight were monitored. **D** Whole liver was sectioned for HE staining. Representative images were obtained at ×200 magnification. The expression of Ki67, PCNA and TGFBR2 was determined by IHC staining. Representative images were obtained at ×400 magnification. **E** As shown in schematic diagram, circSEC24A acts as the ceRNA of miR-606 to regulate TGFBR2 expression and promotes the progression of pancreatic cancer

To further investigate the regulatory effect of circSEC24A on tumor growth in vivo. We firstly constructed the xenograft tumor model in nude mice with or without subcutaneous injection of the treated cells. Next, to compare the proliferation rate of circSEC24A silenced group with control group, the volume of tumors developed in all nude mice was measured and recorded weekly. After 4 weeks, the tumor was removed and weighed. Our data showed that tumor weight in the circSEC24A silenced group was significantly lower than that in the control group (Fig. 7A–C). HE staining showed large and hyperchromatic nuclei in the control group compared with the circSEC24A knockdown group. The control cells were significantly more malignant than circSEC24A knockdown group. Furthermore, IHC staining demonstrated that knockdown of circSEC24A remarkably reduced the expression of Ki-67, PCNA and TGFBR2 in xenograft tumor tissues (Fig. 7D). Taken together, these results suggest that circSEC24A can promote the expression of TGFBR2 through miRNA sponge effect, thereby enhancing the tumorigenicity of pancreatic cancer (Fig. 7E).

## Discussion

Pancreatic cancer is one of digestive cancers with the highest mortality rate [14]. Most cases of pancreatic cancer are generally diagnosed in advanced stages, with poor treatment outcomes and poor prognosis [15]. Emerging evidence has confirmed that circRNAs participated in irreplaceable regulatory functions involved in multiple biological processes, especially in the occurrence, development and metastasis of cancers [16]. CircRNAs have potential to become biomarkers for cancer treatment and diagnosis. Compared with other non-coding RNAs, circRNAs show their unique characteristics, relatively stable and specific expression in cells and tissues [17]. Different circRNAa may function as tumor suppressors or promotors in different cancers. Han et al. reported that circ_0071036 was significantly correlated with unfavorable characteristics and prognosis in pancreatic cancer, and highly expressed circ_0071036 facilitated tumourigenesis [18]. In addition, circBFAR accelerated cell proliferation and invasion of pancreatic cancer via upregulating mesenchymal–epithelial transition factor in pancreatic cancer [19]. Conversely, circNFIB1 suppressed lymphangiogenesis and lymphatic metastasis through inhibiting the PI3K/AKT pathway in pancreatic cancer [20].

In this study, we identified an unreported circular RNA in pancreatic cancer, circSEC24A, with 252 nucleotides in length, which was derived from back-splicing the SEC24A mRNA. Previous studies on SEC24A showed that it exerted as an essential mediator to promote endoplasmic reticulum (ER) stress-induced cell death [21]. Moreover, phosphorylated and stabilized SEC23B interacted with SEC24A and localized to the ER-Golgi intermediate compartment to control autophagy [22]. However, circSEC24A lacked the capacity to encode protein and was reported to function as an oncogene in cutaneous squamous cell carcinoma. The underlying molecular mechanism was that circSEC24A competitively binding miR-1193 to regulate MAP3K9 [23]. In addition, circSEC24A also induced apoptosis and inflammation in chondrocytes [24]. In our data, we found circSEC24A was dramatically high expression in pancreatic cancer tissues and cells. In addition, overexpressed circSEC24A promoted pancreatic cell proliferation, migration and invasion. Subsequently, we utilized bioinformatic method combined with experiments, revealing that circSEC24A exerted as a sponge for miR-606 to modulate TGFBR2, which further activated AKT signaling pathway. These results suggested that circSEC24A served as potential oncogene to facilitate pancreatic cancer progression.

The transcripts of CircRNAs and mRNAs have the same miRNA binding sites, they form a complex to participate in regulate post-transcriptional levels of each other by functioning as ceRNA [25]. The researches about circRNAs mainly focused on their roles in cytoplasm, where circRNAs exerted as sponges for miRNAs, interacted with RNA binding protein to participate in regulatory function in protein translation [26]. Accumulated evidence has reported that most of circRNAs contained MREs and ceRNA mechanism was the prominent route to exert their regulatory functions in biological processes [27]. For example, silencing circFOXK2 significantly inhibited pancreatic cancer progression and elevated miR-942 expression, eventually indirectly suppressed expression of PAX6 and GDNF [28].

TGFBR2, namely TGF-β type II receptor, belongs to a member of the TGF-β signaling, which is involved in tumorigenesis and tumor procession. The TGFBR2 signals participate in cell growth, differentiation, angiogenesis and metastasis [29]. In addition, TGFBR2 binds with ligand and interacts with TGFBR1 to construct a hetero-tetrameric complex, activating smad2 and smad3 to interact with smad4. Eventually, the smad members translocate into nucleus to associate with transcription factors to regulate target gene expression [30]. Moreover, TGF-β signals exist crosstalk with AKT signaling pathway, thereby activated TGF-β could exert promoting function of cancer. Moreover, Previous study has reported TGFBR2 inhibitor as a potent drug for pancreatic cancer treatment, the TGFBR2 inhibitor, Galunisertib significantly improved overall survival in patients with unresectable pancreatic cancer [31]. Our study revealed that circSEC24A could regulate the progression of pancreatic cancer in a TGFBR2-dependent way. However, there are several limitations to our results. Firstly, even if our study confirms the ability of circSEC24A to bind miR-606, it cannot be ruled out that other miRNAs may also bind circSEC24A to regulate the progression of pancreatic cancer. In addition, whether circSEC24A regulates the progression of pancreatic cancer through other mechanisms, such as its interaction with RNA binding proteins, needs further study. Therefore, it is necessary to further understand the therapeutic potential of circSEC24A in pancreatic cancer.

## Conclusions

Up to now, this is the first deeply study to investigate the expression, regulation and function of circSEC24A in pancreatic cancer. Our data demonstrates that circSEC24A competitively binds to miR-606, in turn regulates expression of TGFBR2, activating AKT signaling to promote cell proliferation, migration and invasion of pancreatic cancer. Our study uncovered the relationship between circSEC24A and TGFBR2 in the malignant progress of pancreatic cancer, considering that TGFBR2 inhibitor was a potent drug for pancreatic cancer, therefore, we deduced that circSEC24A/miR-606/TGFBR2 signal axis might become a biomarker for diagnosis and treatment of pancreatic cancer.

## Supplementary Information

**Additional file 1.** Raw data for circRNAs profiling along with fold change in expression and P value.

## Data Availability

All data generated and analysed during this study are included in this published article are available on request.
